# Correction: Filtered beauty in Oslo and Tokyo: A spatial frequency analysis of facial attractiveness

**DOI:** 10.1371/journal.pone.0229523

**Published:** 2020-02-18

**Authors:** Morten Øvervoll, Ilaria Schettino, Hikaru Suzuki, Matia Okubo, Bruno Laeng

References 77, 78, and 79 are incorrect. The correct references are:

77. Mensen A, Khatami R. Advanced EEG analysis using threshold-free cluster-enhancement and non-parametric statistics. NeuroImage. 2013; 67: 111–118. doi: doi.org/10.1016/j.neuroimage.2012.10.027

78. Pernet CR, Latinus M, Nichols TE, Rousselet GA. Cluster-based computational methods for mass univariate analyses of event-related brain potentials/fields: A simulation study. Journal of Neuroscience Methods. 2015; 250:85–93. doi: dx.doi.org/10.1016/j.jneumeth.2014.08.003

79. Groppe DM, Urbach TP, Kutas M. Mass univariate analysis of event-related brain potentials/fields I: A critical tutorial review. Psychophysiology. 2011; 48(12).:1711–1725. doi: doi.org/10.1111/j.1469-8986.2011.01273.x
